# Multiple Roles of the Y Chromosome in the Biology of *Drosophila melanogaster*

**DOI:** 10.1100/tsw.2010.168

**Published:** 2010-09-01

**Authors:** Roberto Piergentili

**Affiliations:** Dipartimento di Biologia e Biotecnologie Sapienza – Università di Roma, Italy

**Keywords:** constitutive heterochromatin, lampbrush-like loops, Homo sapiens Y chromosome, triplex, evolution, Drosophila miranda

## Abstract

The X and Y chromosomes of *Drosophila melanogaster* were the first examples of chromosomes associated with genetic information. Thanks to the serendipitous discovery of a male with white eyes in 1910, T.H. Morgan was able to associate the X chromosome of the fruit fly with a phenotypic character (the eye color) for the first time. A few years later, his student, C.B. Bridges, demonstrated that X0 males, although phenotypically normal, are completely sterile. This means that the X chromosome, like the autosomes, harbors genes that control several phenotypic traits, while the Y chromosome is important for male fertility only. Notwithstanding its long history – almost 100 years in terms of genetic studies – most of the features of the Y chromosome are still a mystery. This is due to the intrinsic nature of this genetic element, namely, (1) its molecular composition (mainly transposable elements and satellite DNA), (2) its genetic inertia (lack of recombination due to its heterochromatic nature), (3) the absence of homology with the X (with the only exception of the nucleolar organizer), (4) the lack of visible phenotypes when it is missing (indeed, except for their sterility, X0 flies are normal males), and (5) its low density as for protein-coding sequences (to date, only 13 genes out of approximately 14,000 have been mapped on this chromosome in *D. melanogaster*, i.e., ~0.1% of the total). Nonetheless, a more accurate analysis reveals that this chromosome can influence several complex phenotypes: (1) it has a role in the fertility of both sexes and viability of males when over-represented; (2) it can unbalance the intracellular nucleotide pool; (3) it can interfere with the gene expression either by recruiting proteins involved in chromatin remodeling (PEV) or, to a higher extent, by influencing the expression of up to 1,000 different genes, probably by changing the availability of transcription factors; (4) it plays a major role (up to 50%) in the resistance to heat-induced male sterility; (5) it affects the behavior; and (6) it plays a role in genetic imprinting. In the present paper, all these Y-related phenotypes are described and a potential similarity with the human Y chromosome is drawn.

[*] I dedicate this paper to my late colleague Nikolaj Junakovic.

## WHAT IS A "GENE"?

The first definition of the word "gene" is as old as the year 1909. Wilhelm Johannsen first used it[1] to name the "inheritable factors" causing variation in visible traits, which had been discovered by the famous experiments of Gregor Mendel[2]. Since then, scientific research has better defined these factors, but, in turn, has also complicated the definition itself of "gene". To date, there is still no agreement about the "exact" meaning of the word "gene"[3,4,5]. Experiments performed by T.H. Morgan in 1910 and by A.H. Sturtevant in 1913, which are summarized in a famous book published in 1915[6], changed the definition of "gene" as a discrete unit of heredity into a physical "locus", since it is possible to map any gene, relatively to the others, in any given chromosome. In 1909, Sir A.E. Garrod first pointed out that many human diseases related to recessive traits are due to metabolic defects[7]. However, only in 1941 did the experiments by Beadle and Tatum on *Neurospora crassa* allow the generalization stating "one gene, one enzyme"[8], which, 15 years later, became a less specific "one gene, one polypeptide"[9,10]. The discovery of the structure of the double helix[11] and the identification of the genetic code[12,13] transformed the definition of "gene" as a "transcribed code", and it was rapidly found that not all genes have proteins as their final output; for example, the genes encoding ribosomal and transfer RNAs are clearly of a different type. Recently, the Sequence Ontology Consortium provided the following definition of gene: "a locatable region of genomic sequence, corresponding to a unit of inheritance, which is associated with regulatory regions, transcribed regions and/or other functional sequence regions"[3,4]. This definition hints at two considerations: (1) especially in the first part, it closely resembles the original definition with the modification introduced by Morgan's studies, indicating that it is difficult to identify a gene by simply looking at the DNA sequence – indeed, although less accurate, a "locatable unit of inheritance" is still a good definition for "gene"; (2) the definition of "gene" is much wider, including functional sequences that do not necessarily include protein-coding sequences and that are not necessarily transcribed. For example, it is possible to include in the cited definition regions of DNA that are important *per se* (e.g., telomeres, centromeres), even when the DNA sequence is highly variable (like it happens for the centromeres), which are not transcribed, but have crucial functions in genome stability.

The Y chromosome of *Drosophila melanogaster* accounts for ~40 Mb of DNA[14,15], but contains only a few protein-coding sequences (to date 13 genes, one of which is in multiple copies) and two RNA-coding genes (i.e., the nucleolar organizer and the *crystal* locus[16]). Extremely long introns characterize some of these genes, but their presence alone is insufficient to explain the DNA content of this chromosome or the reason of their length. So the role of the majority of the heterochromatic regions contained in the Y chromosome is still to be elucidated. Moreover, most animal Y chromosomes are alike, e.g., (1) they are predominantly heterochromatic, (2) they do not possess a number of protein-coding genes comparable to similarly sized autosomes from the same organism, and (3) they are enriched in low-complexity DNA sequences. Therefore, it is likely that this kind of DNA plays some role and is not clusterized there just by chance, even if Y chromosomes may originate in several different ways[15]. Classic genetics was able to deal only with the few known sequences cited above, probably because most of the DNA content of the Y chromosome does not have a "classic" role. Even nowadays, the gene definition is a debated topic. This suggests that we should change the way we look at, and consequently study, these sequences. The Y chromosome is almost certainly not just a collection of a few genes interspersed in "junk DNA", but a chromosome that is important *per se*, whose presence alone (in some cases independently of the housed genes) may influence the behavior of many other genes located elsewhere in the genome in a continuous way. Also, the evolutionary role of this chromosome is important, especially in the creation of species-specific barriers and as a molecular clock for the studies of divergent speciation. In this perspective, *D. melanogaster* is once again a suitable model organism for the evolutionary and functional study of this type of DNA.

## ON THE ORIGIN OF Y CHROMOSOMES AND THEIR ROLE IN SPECIATION

It is generally accepted that the sex chromosomes of most animals arose from a couple of homologous autosomes after the acquisition of a dominant sex determination gene[17,18] and that the Y chromosome is the result of the degeneration of one of them[19,20,21]. Its degeneration would be a consequence of its lack of recombination with the X[22,23,24,25]. It is possible that this nonselective situation favors the maintenance of repetitive DNA[26], although 40 years ago, a theory was proposed for a possible functional role of this type of sequence[27]. Interestingly, this theory was later supported by new studies on the mouse B2 transcript involved in heat-shock-induced transcription inhibition[28,29]. In general, the Y chromosome of most animals shows two features: (1) it harbors and accumulates by various means, mainly transposition and chromosome translocations, genes involved in male fertility[30]; and (2) most of its genes tend to degenerate rapidly, so that only a few of them remain functional[31]. Nonetheless, at least to some extent, pseudogenes also seem to evolve under functional restriction, so that they may gain new functions during evolution[32,33]. The evolution of the Y chromosome can be very fast, as recently reported for *D. miranda*[34]. This model system is particularly suitable for studies of this kind. Approximately 1 million years ago, an autosome of this fly fused to the Y chromosome[35], forming a so-called "neo-Y". The nonfused homologue is present in one copy in males and in two copies in females, and for this reason it is called "neo-X". Since in these flies, as in *D. melanogaster*, males completely lack meiotic recombination, and since the neo-Y is transmitted only from fathers to sons, the neo-X and neo-Y are completely unable to exchange genetic information by crossing over. A similar event probably also occurred for the human Y chromosome around 100 million years ago[36]. Comparative molecular analysis of the neo-sex chromosomes of *D. miranda* shows that transposable element insertions are relatively rare in the neo-X (<1%) and far more abundant in the neo-Y, for which about 20% of the chromosomal DNA is either a transposon or is transposon derived. Moreover, indirect evidences suggest that overall repetitive sequences, including satellite DNA, might account for up to 50% of the neo-Y chromosomal DNA in *D. miranda*[34]. Finally, the same study underlines that almost half of the genes on the neo-Y accumulated mutations, such as early stop codons or frameshifts, which impair their function at the protein level. Nonetheless, functional genes on the neo-Y are still present, but seem to evolve more slowly than the average, and this is also true for their homologues on the neo-X. Transposons might play a role in gene regulation in the Y chromosome as well, being able to silence gene expression locally[34]. In *D. melanogaster,* the accumulation of male-related genes on the Y has been reported, but differently from *Homo sapiens,* the homologous genes are located on the autosomes, not on the X[15]. Even more striking is the discovery that Y chromosomes from closely related drosophilids may be very different[15,37], thus leading some authors to define the Y chromosome as "a chromosome that regularly segregates from the X chromosome, irrespective of its origin"[15]. This suggests two considerations: (1) although all Y chromosomes seem to evolve similarly in many organisms once they have been established, their origin may be very different even in the same taxon[15,38], being derived either from a couple of autosomes or from some B chromosome, or from both[14,15,39,40]; (2) the Y chromosome patroclinous inheritance favors the accumulation of genes and functions related to male fertility – whatever chromosome they originally came from – so that these genes are under evolutionary pressure like genes on recombining chromosomes. Notably, this accumulation is relatively fast, being ~11 times higher than the gene loss due to spontaneous mutations and probably accounts for the different gene complement of the Y chromosomes of various drosophilids[15,37].

Among the genes mapping in the Y chromosome, it is possible to find loci involved in reproductive isolation and, therefore, this chromosome also exerts a role in this phenomenon. Indeed, it has been demonstrated that hybrid male sterility is the major cause of lethality or sterility in crosses between different species. In particular, the genes involved in this event are at least ten times more numerous than those causing similar phenotypes in females[41,42,43,44] and this process is also evolutionarily very fast[41,45,46]. One of these genes, called *Odysseus*[47,48] has been recently reported as one of the main causes of male sterility in crosses between *D. mauritiana* and *D. simulans*, causing extensive constitutive heterochromatin decondensation in the Y chromosome of *D. simulans*[49]. Interestingly, this is not the only case in which heterochromatin is involved in hybrid sterility – a similar situation occurs in *D. melanogaster* for the *Zhr* locus[50,51], a satellite DNA located in the X heterochromatin which interferes *in cis* with the normal chromosome segregation[52] and with the heterochromatin binding protein Lhr[53].

## THE Y CHROMOSOME OF *D. MELANOGASTER* AND ITS GENETICS

The nature of the Y chromosome has been an obstacle for its genetic analysis. Morgan demonstrated that this chromosome does not contain the same genes of its "homologue" (the X) and it is impossible to map genes on it because it shows almost no recombination[54,55,56,57,58,59,60]. Also, mutagenesis studies failed; the Y chromosome does not contain genes whose function is evident in phenotypic traits and it does not harbor genes essential for viability. Indeed, aneuploid flies lacking it, with an X0 karyotype, are viable males[61,62] and only show a weak thinning and shortening of bristles and sometimes etching of the abdomen. We now know that these visible phenotypes are mainly due to the reduced number of rRNA-coding genes, which are located in the constitutive heterochromatin of both sex chromosomes[54,63,64,65,66,67,68,69] and are responsible for X-Y pairing during the male meiosis[70]. The Y chromosome was also demonstrated to be genetically inert[71] and entirely heterochromatic[72,73,74]. It comprehends approximately 12% of the male genome[75] for a total of ~40 Mb of DNA[14,15], and is composed mainly of transposons and repetitive, simple sequence DNA (Fig. 1 and references therein). However, a clear phenotype is associated with the Y chromosome: X0 males are completely sterile and the factors responsible for this trait are located on both chromosomal arms[54]. Sex determination and dosage compensation in *D. melanogaster* are different from mammals in general and *H. sapiens* in particular[90]; in fact, in the fruit fly, they are both driven by the ratio between the X chromosome(s) and the autosomes. Thus, both XY and X0 flies are males (but the latter are sterile), and both XX and XX-Y flies are females (in both cases, they are fertile). Consequently, XX-Y females may be crossed to obtain offspring with supernumerary Y chromosomes, such as XY-Y (fertile) males, which in turn may be crossed as well to obtain other, more complex karyotypes. This allows the analysis of the influence of the Y chromosome in a variety of genetic backgrounds. The analysis of the hyperploidy of the Y chromosome in either males or females indeed showed some unexpected, yet very interesting features. The Y chromosome exerts an epigenetic effect in the PEV (position effect variegation) phenomenon[91] in the way that an extra Y in the karyotype (XX-Y females and XY-Y males) suppresses the variegation[92,93,94,95,96,97]. On the contrary, a missing Y (X0 males) enhances PEV[98]. Hyperploid XX-Y females also show a modification of the nucleotide pool balance inside unfertilized eggs, although none of the genes mapped on the Y chromosome to date seem to be involved in nucleotide metabolism. In particular, eggs show a decrease of thymine and a contemporary increase of cytosine, although the total amount of pyrimidines remains the same if compared to eggs produced by XX females; instead, the overall content of purines is doubled, mainly due to adenine increase[99,100]. Further, raising the number of Y chromosomes causes new phenotypes to occur: (1) mottling of the eyes in both sexes[101] and male sterility[101,102] for two extra Y chromosomes (i.e., XX-Y-Y females and XY-Y-Y males); (2) perhaps female sterility and male lethality for three extra Y chromosomes (XX-Y-Y-Y females and XY-Y-Y-Y males)[103,104].

## MAPPING AND IDENTIFICATION OF GENES IN THE Y CHROMOSOME OF *D. MELANOGASTER*

In order to better understand the role of the sex chromosome in male fertility, Brosseau performed an extensive genetic analysis of Y chromosome rearrangements that allowed him to localize seven loci, five on the long and two on the short arm[81]. They were named *kl-1* to *kl-5* on the long arm, and *ks-1* to *ks-2* on the short arm. However, subsequent studies did not allow the identification of the *kl-4* gene, so for many years it was assumed that only six genes – or, better, "fertility factors" – were located on

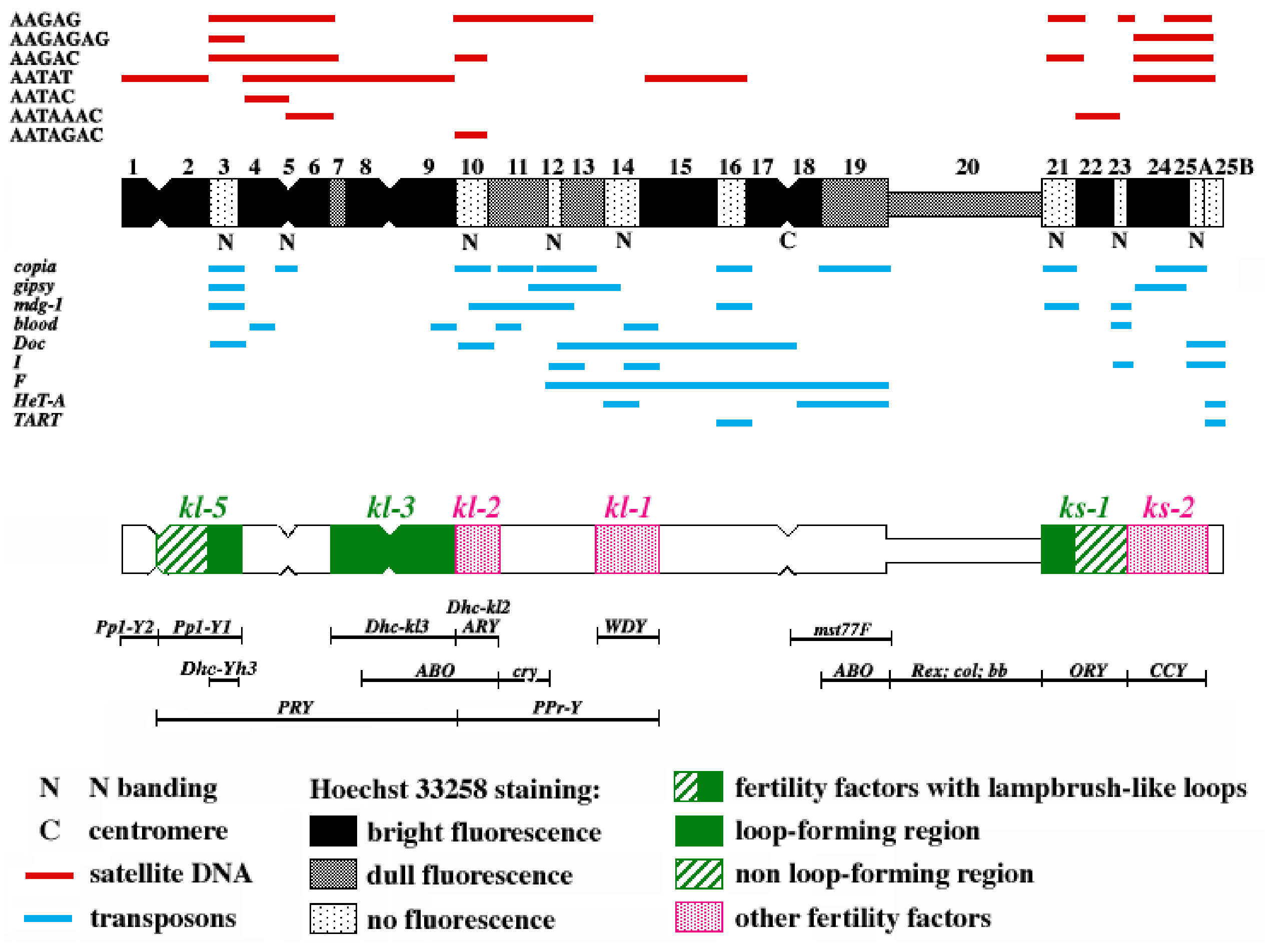

**FIGURE 1.** Schematic representation of the Y chromosome of *D. melanogaster*. All parts of the scheme are aligned with the Hoechst 33258 banding. From top to bottom: cytological localization of satellite DNAs[76,77]; Y chromosome diagram showing N banding and Hoechst 33258 banding[75,78]; cytological localization of transposable elements[79,80]; Y chromosome diagram showing the cytological localization and organization of six[78] of the fertility factors described by Brosseau[81]; other genetic elements mapping on the Y chromosome[78,82,83,84,85,86,87]. (Note on the image: this picture is reprinted from Piergentili and Mencarelli[88] and modified according to Vibranovski et al.[86]; no data are available to date about the location of the fdy gene[89].)

it[78,81,105,106,107]. Their fertility function is exerted mainly during the development and maturation of primary spermatocytes[108,109] and their absence or malfunction causes several problems during spermatogenesis (Table 1), finally leading to the development of immotile sperms.

The analysis of Y chromosome rearrangements demonstrated that each fertility factor spans ~4,000 kb of DNA, so it is more than 100 times larger than the average eukaryotic locus. Three of these fertility factors – namely, *kl-3*, *kl-5*, and *ks-1* – are responsible for the formation of giant lampbrush-like loops, which recall the lampbrush chromosomes of amphibian oocytes, from which they also take their name[107]. These “Y loops” are the cytological manifestation of genetic activation of the Y chromosome during the primary spermatocyte development[110] and are a conserved feature of many drosophilids[111]. Interestingly, during the meiotic metaphases, the sister chromatids of the Y chromosome are separated, which is cytological evidence of their previous activity; instead, in all other tissues, the sister chromatids are close to each other and indistinguishable in mitotic metaphases because of the “stickiness” of the heterochromatin (Fig. 2). The *kl-3* loop at its maximum extension appears as a thin filament with no free ends when analyzed at the phase contrast microscope, while the other two loops have a more dense aspect[110], although they probably have a thread-like organization as well[88,112].

**TABLE 1**
**Fertility Factors Missing from the Y Chromosome and Corresponding Abnormal Phenotypes in *D. melanogaster* Spermatogenesis**

| Missing Fertility Factor | Premeiotic Defects | Postmeiotic Defects |
|---|---|---|
| *kl-1* | None described | Wrong nebenkern/axoneme alignment; missing tubular structures inside cyst lumen; wrong coiling of sperm tails; wrong spermatid orientation; missing spermatids inside the cyst; immotile sperm tails |
| *kl-2* | None described | One high-molecular-weight polypeptide missing (~350 kD) |
| *kl-3* | *kl-3* loop absent | Absence of external dynein arms from axoneme peripheral doublets; one high-molecular-weight polypeptide missing (~300 kD); wrong nebenkern/axoneme alignment |
| *kl-5* | *kl-5* loop absent | Absence of external dynein arms from axoneme peripheral doublets; one high-molecular-weight polypeptide missing (~300 kD); wrong individualization of spermatids; nebenkern without axonemes and vice versa; partial cytoplasm retention inside sperm heads; immotile sperm tails |
| *ks-1* | *ks-1* loop absent | Missing tubular structures inside cyst lumen; wrong nebenkern/axoneme alignment; similar, but stronger, spermatid defects compared to *kl-1*; sperm tail coiling apparently normal, but with immotile sperm tails |
| *ks-2* | None described | Wrong nebenkern/axoneme alignment; failures in the individualization of spermatozoa; nebenkern without axonemes and vice versa |

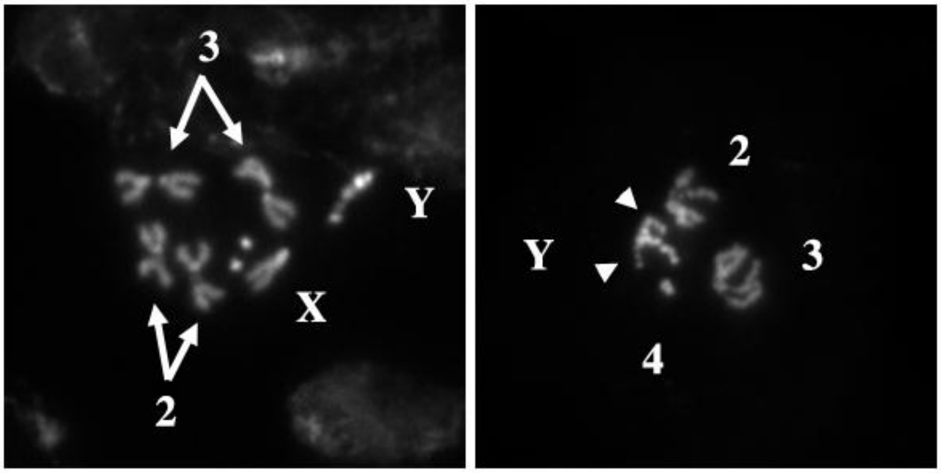

**FIGURE 2.** Comparison between mitotic (left) and meiotic (right) Y chromosome in the male germ line of *D. melanogaster*, as they appear with the DAPI dye. Numbers indicate chromosomes (on the left image, chromosomes number 4 are the white dots next to the X chromosome). (Left) mitotic metaphase from a larval neuroblast. Note that the entirely heterochromatic Y chromosome shows attached sister chromatids as a consequence of the "stickiness" of the heterochromatin. (Right) A meiotic metaphase II. Note that the Y chromosome, recognizable by chromosomal banding, shows separated sister chromatids (arrowheads) as a remnant of its genetic activation during the primary spermatocyte development. (Note on the image: original microphotographs of the author.)

The development of "Y loops" is temporally controlled: the *kl-3* loop is the last to form, and all loops rapidly disintegrate during the late meiotic prophase I[110], probably as a consequence of chromosome condensation. These loops are also characterized by their ability to bind large amounts of proteins; they are intensely stained by the Giemsa, Coomassie blue, and dansyl-chloride dyes[110]. Many bound proteins have been isolated and characterized, and antibodies have been raised against some of them[111,113,114,115,116,117,118,119,120,121,122,123,124,125]. It is interesting to note that none of these proteins is encoded by genes mapping on the Y. Also, their function is highly variable – DNA- and RNA-interacting proteins, testis-specific proteins accumulated either in the nucleus or in the sperm tail. Other than the six fertility factors and the nucleolar organizer, more functions were assigned to the Y chromosome during the years (Fig. 1 and Table 2)[78,82,83] but, as expected, the genetic analysis of these genes has been very difficult. The discovery of new genes had a stop until the completion of the *D. melanogaster* genome sequence analysis[126] offered new tools to solve this problem. Computer-based approaches[127] allowed the discovery of more protein-coding sequences on the Y[84,85,86,87,89], thus increasing the number of "classical" genes (either protein- or RNA-coding) to at least 15[14,15] (Fig. 1 and Table 2). Notably, many of these genes have at least one autosomal, but not an X-linked, counterpart, so it is not clear yet if their activity, if any, is necessary or just redundant[15,84,85,86,87]. It is worth underlining that all six "fertility factors" described in 1960 are now correlated to the presence of at least one protein-coding sequence (Fig. 1 and Table 2), thus strongly suggesting that the impaired male fertility of the translocated Y chromosomes studied by Brosseau is due to the interruption of one of these genes. However, for some of these proteins, an essential role in male fertility was excluded[84,85,86,87] either for the presence of mutations or for the presence of an homologous counterpart, or even because every fertility factor most likely contains only one essential protein-coding gene[105]. Moreover, the reason why only three of these loci have a visible phenotype inside primary spermatocyte nuclei (Y loops) and the others do not is still a topic of discussion (see the end of the next section).

## ROLE OF THE Y CHROMOSOME IN THE HEAT-INDUCED MALE STERILITY PHENOMENON

More than 80 years ago, it was first demonstrated that flies reared at high temperatures do not reproduce because of a reversible sterilization of males[128,129]. For *D. melanogaster*, this occurs at ~30°C; at 32°C, flies die at the pupal stage[130]. Further studies demonstrated that this is a general feature of most, if not all, drosophilids[131,132]. The difference between the temperatures at which lethality or viability/fertility occurs is (1) usually very small (no more than two degrees), (2) species and population specific, and (3) correlated to the average temperature at which wild flies usually live[132]. It is possible to increase the temperature at which flies become sterile by artificial selection[133,134,135]. This selection is possible within a limited number of generations, indicating that this process is very fast by the evolutionary standpoint. Of great interest is the finding that the Y chromosome is directly involved in heat resistance and accounts for at least 50% of the variability[132]. Introducing a Y chromosome of a tropical population in a temperate population by crosses has the effect to increase the heat resistance of the temperate group; on the contrary, the reverse crosses lower the heat resistance of tropical groups[135]. As previously noted, the heat-induced sterility is a reversible phenomenon; bringing males back to the permissive temperature allows the recovery of male fertility in a variable number of days. This number depends on different variables: (1) the time spent at the nonpermissive temperature (the more time spent, the more time needed for the recovery), (2) the temperature itself (recovery after exposure to 31.5°C requires more time than a time-equivalent exposure to 31°C), and (3) the origin, tropical or temperate, of the population considered (tropical populations recover faster)[132,135,136,137,138]. The introduction of an external Y chromosome allows varying significantly the recovery time in all conditions[135]. This clearly indicates that Y-dependent, temperature-related male fitness is a target for natural selection. Moreover, the high speed of evolution of these traits fits quite well with the high variability of noncoding sequences inside the Y chromosome. At the cytological level, male infertility is due to a series of abnormalities in the germ line, such as shorter cyst length and anomalous cyst elongation; spermatid death rate, and, consequently, number of mature sperm heads; and level of chromatin condensation inside sperm heads[132,135]. Interestingly, some of these features are also common in mutant lines having the rearranged Y chromosomes described by Brosseau[81] and missing some of the fertility factors (Table 1) [139].

**TABLE 2**
**Summary of the "Locatable Units of Inheritance" Mapping on the Y Chromosome***

| Unit Name | Unit Type | Location | No. of Copies | Molecular Function | Biological Function |
|---|---|---|---|---|---|
| Pp1-Y2 | Protein coding | h1 | 1 | Serine/threonine phosphatase | Unknown |
| Pp1-Y1 | Protein coding | h2-h3 | 1 | Serine/threonine phosphatase | Unknown |
| PRY | Protein coding | h2-h9 | 1 | Unknown | Unknown |
| Dhc-Yh3 | Protein coding | h2-h3 | 1 | Dynein heavy chain | Sperm movement |
| *kl-5* loop | Structural element | h3 | 1 | Unknown | Triplex formation |
| *kl-3* loop | Structural element | h7-h9 | 1 | Unknown | Triplex formation |
| Dhc-kl3 | Protein coding | h8 | 1 | Dynein heavy chain | Sperm movement |
| ABO | Unknown | h10-h11 and h19 | 2 | Unknown | Suppression of abo mutation |
| Dhc-kl2 | Protein coding | h10-h11 | 1 | Dynein heavy chain | Sperm movement |
| ARY | Protein coding | h10 | 1 | Aldehyde reductase | Unknown |
| cry/Su(Ste) | RNA coding | h11 | ~80 | Protein kinase regulator | Suppression of Ste gene |
| Ppr-Y | Protein coding | h10-h14 | 1 | Protein phosphatase type 1 regulator | Unknown |
| WDY | Protein coding | h14 | 1 | Unknown | Unknown |
| Centromere | Structural element | h17-h18 | 1 | Spindle interaction | Chromosome segregation |
| Mst77F | Protein coding | h18-h19 | 16–20 | Unknown | Unknown |
| bb/col/Rex | RNA coding | h20 | ~200 | rDNA | Protein biosynthesis |
| *ks-1* loop | Structural element | h21 | 1 | Unknown | Unknown |
| ORY | Protein coding | h21-h23 | 1 | Unknown | Unknown |
| CCY | Protein coding | h24-h25A | 1 | Unknown | Unknown |
| Telomere | Structural element | h1 and h25B | 2 | Chromosome ends protection | Chromosome segregation |
| fdy | Protein coding | nd | 1 | Unknown | Unknown |

* According to the text, structural functions are also listed. Data partly collected from FlyBase (URL: http://www.flybase.org) Release FB2010_05.

By performing time measurements for fertility recovery and knowing that at 25°C the spermatogenesis lasts approximately 10 days, it was found that in the male germ line, the most heat-sensitive cells are primary spermatocytes[140], which are genetically characterized by the Y chromosome activation (Fig. 2) and cytologically distinguished by the presence of the Y loops. An analysis of the satellite sequences harbored in these chromosomal regions (Fig. 1) suggests that at least some of them might organize an atypical three-dimensional conformation known as "triplex"[141]. Indeed, the immunostaining of primary spermatocyte nuclei with antitriplex antibodies[142,143,144,145,146,147] revealed that at least two of them, *kl-3* and *kl-5*, are specifically decorated[88]. A study of testes from males reared at the nonpermissive temperature of 31 ± 0.5°C showed that, although apparently normal, the Y loops are no more immunostained by the antitriplex antibodies, suggesting that these structures are heat sensitive[88].

The contemporary absence of triplex staining and of two high-molecular-weight polypeptides associated with the presence[148] and with normal development[149] of *kl-3* and *kl-5* loops for the first time allowed establishment of a correlation among Y chromosome satellite DNA, unusual DNA structures, heat sensitivity, and an abnormal biochemical pattern of proteins involved in the normal sperm tail development[148] and, consequently, in the male fertility. Another set of experiments aimed at the isolation of autosomal mutations affecting the size and/or development of Y loops further supports the possibility that these regions might fulfill a structural role before meiosis. Ceprani and coworkers[150] isolated two mutations, named *dolly-1* and *dolly-2*, whose cytological phenotype is not a reduction or a someway-altered development of the loops, but is their splitting into two identical halves. Since each loop is formed equally by the two sister chromatids (indeed, there is no reason to believe the contrary), it has been suggested that the two halves are the cytological manifestation of some missing chromatin structure at the ultrastructural and/or molecular level, which terminally results in the loop splitting.

Since no other defects are evident in the germ line of mutant flies except immotile sperm tails, the conclusion drawn is that there is a relationship between the ultrastructural organization of the Y loops and the normal development of the sperm tail. The presence of duplicated loops also allows the exclusion of the missing transcription of the open reading frames inside these loci as a cause of sperm immobility, since this part of the Y chromosome is still cytologically – and, probably, genetically – active. As noted before, several proteins encoded by the autosomes localize on the Y loops, so it is likely that these structures are not just the cytological manifestation of the transcription of unusually long genes[151,152,153]. Indeed, these structures may also be places for docking or storing proteins that are ready since before the meiosis, but that will exert their function much later, during the spermiogenesis. While it is not yet clear if this localization also implies some post-translational modification, it is noteworthy that some of the mutant lines with a reduced or absent *kl-3* loop still produce the high-molecular-weight polypeptides described by Goldstein and collaborators[148], although these proteins are unstable and rapidly degraded[149]. This degradation is not an intrinsic feature of such proteins, since they are still visible in testes dissected and kept in physiological solution at 32 ± 0.5°C for 2 h before analysis[88]. Since even reduced loops of mutant flies are still able to bind proteins and are decorated by specific antibodies[149,150], it is likely that this issue is not qualitative, but quantitative. This observation further supports the idea that the open reading frames hosted on the Y chromosome are only partially or not at all involved in the male sterility caused by the above-mentioned autosomal mutations.

## THE Y CHROMOSOME PLAYS A CENTRAL ROLE IN GENOME FUNCTION

In their paper on the male sterility of flies at extreme temperatures, David and collaborators[132] hypothesize two ways to explain the correlation between viability and sterility thresholds. Either these two traits are independent, and for some reason under temperature-driven selection, or they share a genetic basis, indicating that genes with pleiotropic effects may influence both phenotypes. Recent data seem to suggest that the truth is somewhere in the middle. As reported previously, introducing a Y chromosome into a different genetic background may greatly influence the heat resistance. This means that, to a great extent, the Y chromosome alone can influence these traits, being at least partially independent from the other chromosomes. Nonetheless, the genetic background is important as well. The analysis of a large number of male sterile mutations characterized by defective Y loops[150] demonstrates that more than half of the loci identified influence both the normal behavior of Y loops and some aspects of meiosis. This finding indicates that these genes induce pleiotropic, deleterious defects in the male germ line upon mutation. It should also be remembered that several autosome-encoded proteins localize on the Y loops before the meiotic prophase I. Taken together, these data suggest that, at least in the male germ line, the Y chromosome is part of a network of genetic and biochemical interactions. These multiple connections had been partially highlighted by some works illustrating the role of the Y chromosome in sperm competition[84] and its strong role on general male fitness[154], as well as in sex ratio distortion[155,156] and in fly behavior[157,158]. Recently, Lemos and coworkers[159] demonstrated that the Y chromosome polymorphism might deeply influence the activity of many genes (estimated in the range 100–1,000) located on other chromosomes[160,161 for comments], other than on itself. Interestingly, the most abundant genes in the list are those connected to male fertility and influenced by heat stresses, and many of these are important for sperm development. Among these over-represented are genes influencing the mitochondria. This is not surprising; very long sperm tails characterize drosophilids. In *D. melanogaster*, spermatozoa are 1.8 mm long (the fly being approximately 5 mm long); in *D. hydei* and *D. bifurca*, they are 23 mm and almost 60 mm long, respectively[109,111,162,163,164]. During spermatogenesis, after meiotic divisions, mitochondria are organized in spherical structures known as "nebenkern". These structures elongate and divide into two symmetric halves between which the sperm axoneme is built; mitochondria then supply the necessary energy for the sperm tail movement. Because the size of the sperm tails is huge, a gene influencing the mitochondrial function will likely influence male fertility. Another feature resulting from the same study[159] is that the affected genes are more dynamic from an evolutionary point of view, since their expression is more polymorphic. It has been noted that the few functional protein-coding genes on the Y chromosome undergo a stronger than average natural selection as time passes[34], and that the number of gene gains is higher than gene losses[15,37]. Then, probably, the destiny of the Y chromosome is not disappearing, as hypothesized[165,166], and, most likely, its degeneration will stop. This evolution would ultimately lead to the specialization of the Y chromosome as a major modulator, but not as an on/off switch, of gene expression in males. This will possibly result in continuous rather than discrete effects on gene expression[160]. Noteworthy, this effect is exactly as it has been found for heat resistance and for Y loops behavior (i.e., mutations with reduced, but not absent loops). The method used by Lemos is basically the same as that of David (i.e., introducing a "new" Y chromosome inside a different genetic background), and the measurements were performed using the microarray technology. Also in this case, a tropical Y chromosome introduced into a temperate genetic background is able to induce misexpression of several genes, some of them being over- and others underexpressed according to the genetic crosses performed.

Another recent progress in the analysis of the genome-wide influence of the Y chromosome was the discovery that this genetic element may influence dosage compensation by imprinting[167]. The imprinting phenomenon had been previously described in the fruit fly[168,169,170], but in those cases, the Y chromosome imprinted genes physically located on itself, irrespective of their origin (either transposed or translocated after chromosomal rearrangements). In many cases, the expression of a reporter transgene (*yellow*, *white*, or both) transposed into the Y chromosome is different if the Y comes either from XY males or from XX-Y females. Similarly, a *yellow* gene (normally X linked) moved onto the Y chromosome by an X-Y translocation may be influenced in its expression in a similar way, even if *yellow* is *de facto* inside a euchromatic region[170], thus indicating that the Y imprinting can influence non-Y sequences. The work of Menon and Meller[167] pushes this phenomenon farther. Dosage compensation in *D. melanogaster* requires the presence of X- and autosome-encoded proteins, whose ratio determines the maleness or femaleness of the embryo[90]. So, apparently, the Y chromosome is not required for this choice. Once the sex is established, males compensate their one-copy X-linked genes by overexpressing them, compared to females[171]. Dosage compensation is achieved by the MSL complex, which includes also two noncoding RNAs (*roX1* and *roX2*) that are encoded by the X chromosome[172]. Only mutation of both *roX* genes causes male lethality, which indicates that these genes are redundant[173]. The authors found that males carrying a paternally derived *roX1 roX2* double-mutant X chromosome and a maternally derived Y chromosome show a dramatically increased survival when compared to males having the same sex chromosomes inherited in the natural way, and that "this effect can be attributed solely to the presence, and parent of origin, of the Y chromosome"[167]. Indeed, the absence of a Y chromosome (X0 males) confers an intermediate survival, which implies that the mere "presence" of this chromosome is as important as its content.

## OF FLIES AND MEN – DIFFERENT YET SIMILAR Y CHROMOSOMES

A brief list of similarities between the Y chromosomes of flies and humans is reported in Table 3. The Y chromosome of *H. sapiens* is 60 Mb long and has a long block of constitutive heterochromatin on the long arm (Yq), spanning ~40 Mb[174]. There are two pseudoautosomal regions at the tips of the chromosome, accounting for about 3 Mb of DNA. Only these regions are able to pair and recombine with homologous sequences located on the X chromosome, so that 95% of the DNA mapping on the Y is male specific[21]. Instead, the male-specific region (MSY) is able to undergo intrachromosomal recombination, a mechanism probably involved both in its self-maintenance[175] and in the observed high Y chromosome mutation rate and structural polymorphism[176]. The euchromatic portion of the male-specific region essentially contains three types of sequences: X transposed, X degenerated, and amplicons[21]. X-transposed sequences are 99% identical to DNA sequences mapping in Xq21 and are probably the result of massive X-to-Y transposition that occurred about 3–4 million years ago[177,178,179]. Interestingly, although these regions account for a total of 3.4 Mb of DNA, only two protein-coding genes map on them, while a high density of interspersed and repeated elements is present[21]. X-degenerated sequences contain single-copy genes or pseudogenes that are homologous to 27 different X-linked genes. Out of them, only 14 are apparently functional; these genes have an X-linked homologue coding for slightly different isoforms[21] and are ubiquitously expressed in 12 cases. Finally, amplicons are repeated, Y-specific sequences spanning a total of 10.2 Mb. They contain most of the genes exclusively expressed in male testes and their organization can be either a direct or an inverted repeat. Amplicons are also the units of more complex chromatin structures called palindromes[180]. Most of the mutations and microdeletions causing male infertility map inside the euchromatic portion of the Y chromosome[181,182,183]; in particular, only 6% of the sterility-causing microdeletions map outside the "azoospermia factor" (AZF) regions[184]. However, the lack of one or more genes is sometimes insufficient to explain the Y-related male sterility, also because of the redundancy of many sequences. In a recent paper[185], the authors hypothesize that the sterility associated with some microdeletions might be due not just to the absence of a gene, but to the alteration of the chromatin structure, thus impairing the premeiotic X-Y chiasma formation. Moreover, other studies demonstrated that the same microdeletion may or may not be the primary cause of infertility[186], indicating that the genetic background may influence the genetic behavior of the Y chromosome[183]. It is interesting to underline that the majority of the human Y chromosome, i.e., the heterochromatic portion, has not been investigated yet for the presence of "functions" related to male fertility, because of the absence of well-characterized chromosome rearrangements, the low complexity of the harbored sequences, and the apparent lack of any protein-coding sequence. However, it has been reported that the subtotal deletion of the Y chromosome is linked at least to morphophysiological variability[187, cited in 183].

The Y chromosomes of flies and humans are clearly different, yet they share interesting features (Table 3). If we look at the relatively few molecular data available, the similarity is even more interesting. For example, the RNA-binding "deleted in azoospermia" (DAZ) protein, encoded by the human Y chromosome and responsible for a high number of Y-related male infertility clinical cases, has a homologue in the fruit fly called Boule. Interestingly, antibodies raised against Boule demonstrated that this protein moves to the *ks-1* loop before *D. melanogaster* male meiosis, indicating that it is able to specifically recognize such structures[121]. Is it possible that the human Y chromosome forms loop-like structures as well? Are there chances that this protein also recognizes similar structures in *H. sapiens*? May the absence of such structures in humans, if they occur, or the inability of a mutated DAZ protein to recognize them, impair male fertility, as it happens in fruit flies? Is it possible that triplexes also have a role in human spermiogenesis? Can we establish a correlation between the morphophysiological variability described in humans and that described in flies for large alterations in the total amount of Y-related constitutive heterochromatin? A closer look at the structure and function of the heterochromatic portion of the human Y chromosome may possibly lead to new, exciting discoveries for the widespread problem of male infertility in humans.

**TABLE 3**
**Similarities between Fly and Human Y Chromosomes***

| Feature | Human | Fruit Fly |
|---|---|---|
| Portion lacking cross-over | 95% | 100% |
| Proportion of heterochromatin | ~65% | 100% |
| Length of heterochromatic sequences | ~40 Mb | ~40 Mb |
| Genes with male-specific expression | Yes | Yes |
| Presence of active transposable elements | Yes | Yes |
| Presence of satellite DNA | Yes | Yes |
| Number of protein-coding genes | 60 | 13 |
| rRNA genes | 7 | ~200 |
| Other RNA-coding genes | 37 | 1 |
| X0 individuals | Viable/sterile | Viable/sterile |
| Evolutionarily conserved proteins | Yes | Yes |

* Data about genes on the human Y chromosome were collected from Ensembl – Release 58 (URL: http://www.ensembl.org/).

## CONCLUSION

The study of the function of constitutive heterochromatin, although started several decades ago, is still in its early stages. For example, centromeres and telomeres are portions of the genome that harbor repetitive DNA sequences, for which a lot of interacting proteins have been described; yet a comprehensive model of function is missing. The situation for the Y chromosome is even worse. Many genetic aspects of the satellite DNA functions are elucidated, but in most cases, there is no molecular model at all to explain how heterochromatin exerts its role. Data collected to date allow us to draw some conclusions: (1) many, if not all, of the influenced characters show a continuous variation, indicating that the role of the Y chromosome is not an on/off switch, but a fine regulator of complex phenotypes; (2) the Y role is not exerted by the "classic" protein-coding genes that it contains since none of them, to date, has been directly related to these phenotypes; and (3) the Y chromosome is able to interact physically with a very large number of autosomal-encoded proteins, as has been demonstrated for the proteins loaded on the Y loops and for its role in PEV. Taken together, these data indicate that, at least in the male germ line, the Y chromosome is part of a network of genetic and biochemical interactions that only recently are beginning to be elucidated.

Most of these features can evolve very quickly, strongly suggesting that the main role is likely exerted by the noncoding sequences – transposable elements and simple-sequence satellite DNA. Instead, the few male-specific coding sequences, mostly involved in sperm tail formation and function, seem to be under stronger than average evolutionary pressure, mainly because they encode proteins that have specific roles in the male germ line. Interestingly, repetitive DNA sequences account for most chromosomal DNA and are a typical feature of all animal Y chromosomes. As a result, despite its lack of recombination and its patroclinous inheritance, the Y chromosome is still subject to natural selection. The discovery that in many cases the noncoding parts of the chromosome also have an important role suggests that this part of the chromatin might be under evolutionary pressure as well. However, it is clear that the forces acting on it and the way the evolution is exerted at the molecular level have to be very different – in this case, as stated before, we are talking about "genes" in the widest meaning. Repetitive DNA changes are essentially quantitative (i.e., are dose dependent) and not qualitative. Indeed, introducing point mutations in a stretch of satellite DNA 4,000 kb long (the size of the loop-forming fertility factors) is simply insignificant for the cell, so we may expect that this DNA mostly influences continuous characters – exactly what was discovered during almost a century of studies. In this perspective, it is clear that this part of the genome cannot be studied using standard genetic or biochemical techniques; this explains why, although important, only a few results concerning how this chromosome influences cell metabolism were obtained by using these methods. It is conceivable that most of the phenotypes related to the Y chromosome are in some way connected to some high-level structures of the chromatin; in this resembling what happens for eukaryotic centromeres and telomeres. The recent discovery of triple-stranded nucleic acids built by two loci on the Y chromosome shows another way this DNA can be organized at the three-dimensional level. This "unusual" structure is quite widespread among eukaryotes and since it is related to the presence of two of the Y loops, it is likely that it is a sort of molecular framework on which several proteins are loaded before meiosis. The discovery that this structure (1) may vary in a continuous way (i.e., mutants show variably reduced, but not absent loops), (2) is heat sensitive, and (3) influences the presence of proteins involved at least in male fertility, further supports the fact that noncoding DNA on this chromosome has a specific and important role in the biology of drosophilids. Heterochromatin is not just a collection of selfish genetic sequences whose only aim is to reproduce themselves from one generation to the next. The fact that these DNA are widely represented in all genomes strongly suggests that they should have a function; if not so, the speed of evolution of the Y chromosome would have caused them to disappear a long time ago.

## ACKNOWLEDGMENTS

I would like to thank Tammy L. Moser (Duke University Medical Center, Duke University, Durham, NC) and Alessandro Porrello (Institute for Genome Sciences and Policy, Duke University, Durham, NC) for careful review of the manuscript.
